# Comparable impact of lymph node metastases in T2 gallbladder cancer on postoperative prognosis irrespective of the extent of the metastases: A retrospective analysis

**DOI:** 10.1002/jhbp.12140

**Published:** 2025-03-24

**Authors:** Yoji Kishi, Teiichi Sugiura, Takashi Mizuno, Hiromichi Ito, Yu Takahashi, Takehiro Noji, Yuta Abe, Shimpei Otsuka, Shoji Kawakatsu, Asayo Kato, Masayuki Tanaka, Tomoki Ebata, Satoshi Hirano

**Affiliations:** ^1^ Department of Surgery National Defense Medical College Tokorozawa Saitama Japan; ^2^ Division of Hepato‐Biliary‐Pancreatic Surgery Shizuoka Cancer Center Shizuoka Japan; ^3^ Division of Surgical Oncology, Department of Surgery Nagoya University Graduate School of Medicine Nagoya Japan; ^4^ Division of Hepatobiliary and Pancreatic Surgery Cancer Institute Hospital, Japanese Foundation for Cancer Research Tokyo Japan; ^5^ Department of Gastroenterological Surgery II Hokkaido University Faculty of Medicine Sapporo Hokkaido Japan; ^6^ Nursing Division Faculty of Health Sciences, Hokkaido University Faculty of Medicine Sapporo Hokkaido Japan; ^7^ Department of Surgery Keio University School of Medicine Tokyo Japan

**Keywords:** gallbladder neoplasms, lymph nodes, lymphatic metastasis, neoplasm staging, prognosis

## Abstract

**Background:**

Lymph node metastases beyond the hepatoduodenal ligament are sometimes encountered in locally limited T2 gallbladder cancer (GBCA). However, the incidence and impact on prognosis remain unclear.

**Methods:**

This was a retrospective multi‐institutional study of patients who underwent surgical resection for GBCA from 2002 to 2022. The eighth edition of the Union for International Cancer Control staging was used for tumor‐node‐metastasis categorization. The lymph node location was classified as follows: (A) along the hepatoduodenal ligament and common hepatic artery; (B) posterior side of the pancreatic head; and (C) others. Metastasis to regions A, B, and C nodes was denoted as Na, Nb, and Nc, respectively.

**Results:**

Data for 379 patients (pT1, 29; pT2, 162: pT3, 141; and pT4, 47) were evaluated; none with pT1 GBCA had node metastasis. For N1/2 GBCA, the proportion of patients with N2 disease increased with increasing T grade (*p* = .001), while the proportions of patients with Na, Nb, and Nc disease were comparable between pT2 (61%, 26%, and 13%), pT3 (63%, 26%, and 12%), and pT4 (50%, 38%, and 12%) disease (*p* = .681), respectively. Overall survival for pT2N1/2 disease (5 years, 43.8%) was comparable to that for pT3/4N0 disease (5 years, 37.2%; *p* = .192). Among patients with node‐positive pT2 disease, overall survival was comparable for Na, Nb, and Nc disease, with 5‐year survivals of 46%, 43%, and 31%, respectively (*p* = .346).

**Conclusion:**

Region B or C node metastasis was not rare even in pT2 GBCA. Regarding survival outcomes, pT2 node‐positive GBCA should be considered advanced disease irrespective of the extent of node metastasis.

## Graphical abstract

Kishi and colleagues found that in node‐positive gallbladder cancer, the incidences of extensive metastases beyond the hepatic pedicle and of metastases skipping cystic nodes were similarly frequent across stages T2, T3, and T4 disease. Postoperative prognosis in T2 node‐positive cases was not affected by the extent of nodal involvement.

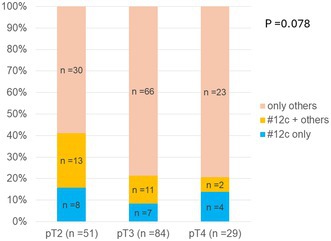

## 1 INTRODUCTION

The prognosis of gallbladder cancer (GBCA) depends on tumor stage, and in particular, the impact of local extension is significant. Organs involved by the tumor differ by tumor location. In the current American Joint Committee on Cancer (AJCC)^1^ or Union for International Cancer Control (UICC)^2^ tumor‐node‐metastasis (TNM) staging system, GBCA with extraserosal invasion is defined as grade T3 or T4, and grading into each category is determined on the basis of the number of involved organs. T3 or T4 tumors invading the hepatic hilum, which frequently leads to obstructive jaundice, are associated with a dismal prognosis, with a reported 5‐year postoperative survival rate of 6%–23%.^3^, ^4^, ^5^, ^6^ However, we sometimes encounter patients with locally limited GBCA, especially T2 disease, but with extensive lymph node metastases. Although the negative impact of lymph node metastases on prognosis has been extensively reported,^7^, ^8^ the prevalence of metastatic nodes specifically in T1/2 GBCA and their impact on prognosis have rarely been evaluated except in a small number of single‐institution studies.^9^, ^10^ So far, the largest series of T2 gallbladder cancer was an international multicenter study, including 937 patients by Kwon et al., showing clear stratification of survival between those with N0, N1, and N2 according to AJCC classification.^11^ However, details of the location of positive nodes were not described in this study. In the current TNM staging systems, the N status is defined on the basis of the number of metastatic nodes.^1^, ^2^ Notably, the lymph nodes along the cystic duct, often denoted as “#12c,” are commonly called sentinel nodes, although their role as a predictor of further node metastases is controversial because node metastases skipping #12c are often encountered.^12^, ^13^ Yasukawa et al. reported that #12c node metastases could be used as sentinel nodes to predict downstream node metastases only in T2 GBCA located on the peritoneal side.^13^ Considering the authors' research, the relation of the sites of metastatic nodes with their number and the postoperative prognosis, especially in patients with T2 GBCA, remains to be addressed.

The purpose of this study was to elucidate the incidence and extent of node metastases and the influence on prognosis after surgical resection in patients with T2 GBCA.

## 2 METHODS

**FIGURE 1:**
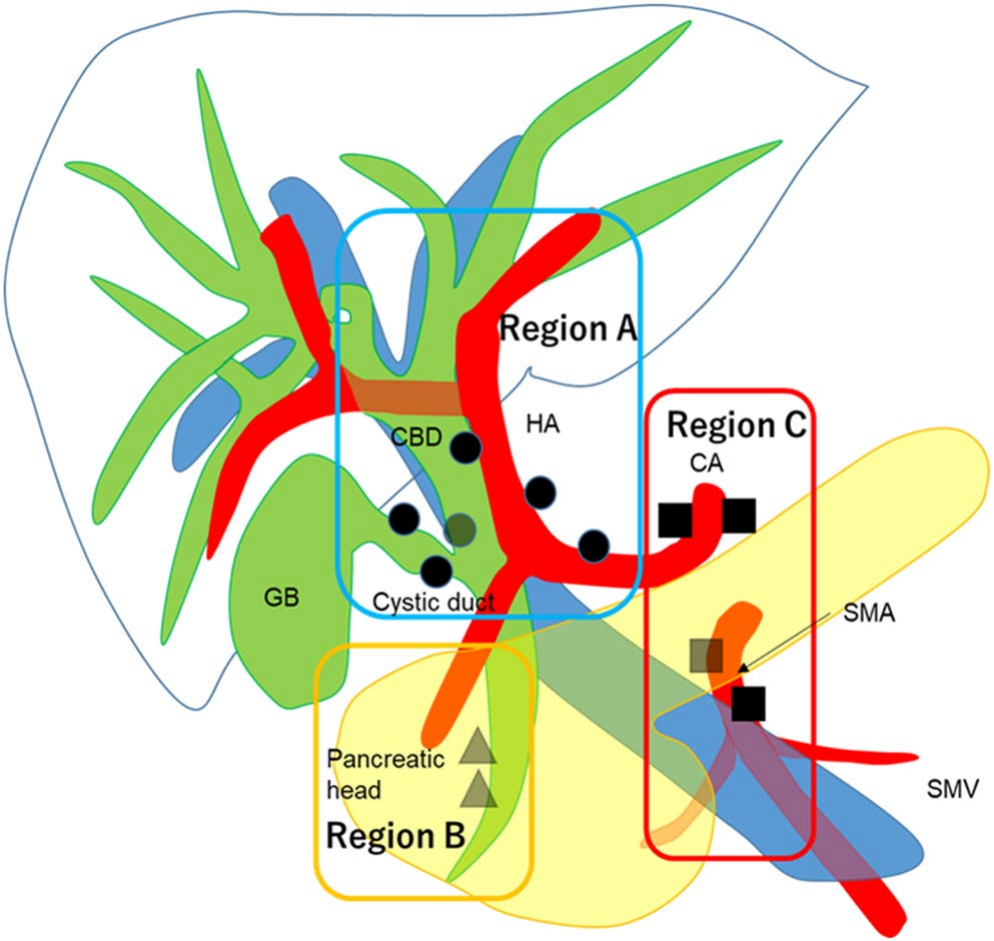
Definitions of region A, B, and C lymph nodes. CA, celiac axis; CBD, common bile duct; GB, gallbladder; HA, hepatic artery; SMA, superior mesenteric artery; SMV, superior mesenteric vein. *Source:* Cited with permission from Kishi et al.^8^

This was a retrospective study using a database created from the medical records in six tertiary referral institutions in Japan. The institutional review board of each institution approved this study (#4718), waiving the need to obtain individual patient informed consent. The database included patients who underwent surgical resection for GBCA from 2002 to 2022. Patients with macroscopically noncurative resection, insufficient data, and pathologically evaluated total lymph node count (TLNC) <6 were excluded. The TLNC ≥6 criterion was based on a proposal by Ito et al. to minimize the underestimation of the prognosis of patients with N0 disease,^14^ and this recommendation was described in the eighth edition of the AJCC guidelines.^1^ The eighth edition of the UICC staging was used to categorize pathologic TNM staging.^2^ Locally limited disease was defined as a tumor of pathologic (p) stage T1/2. However, the database did not include full information on the tumor location on the gallbladder wall; therefore, T2a and T2b were not discriminated. Regarding the N status, N0, N1, and N2 were defined as 0, 1–3, and ≥ 4 regional lymph node metastases, respectively. The location of the metastatic lymph nodes was also recorded. The extent of regional lymph node metastasis was defined according to a previous report as follows: region A, along the hepatoduodenal ligament and common hepatic artery; region B, posterior side of the pancreatic head; region C, others, including the regions along the celiac axis and superior mesenteric artery. Metastases limited to the nodes of region A, region B with or without region A, and region C with or without region A/B were denoted as Na, Nb, and Nc, respectively (Figure 1).^8^ Generally, region A, B, and C corresponded to the extent of regional node metastasis defined by the AJCC,^1^ Japanese staging,^15^ and UICC,^2^ respectively,^8^ but for this study, any nodes outside of region A or B were included in region C. The standard procedure for GBCA was local resection of the tumor securing sufficient surgical margins and dissection of regional lymph nodes defined by the Japanese staging (region A and B). Extrahepatic bile duct resection was indicated when the preoperative diagnosis suggested direct tumor invasion, when lymph node metastases along the hepatoduodenal ligament were suspected, or when the frozen section of the cystic duct stump was positive for cancer invasion.

First, the proportion of patients with node metastases based on the number and sites was compared between each T category of GBCA. To evaluate the extent of nodal metastases, the incidence of metastases skipping the nodes along the cystic duct (#12c) or the nodes in region A was also evaluated. Second, we evaluated postoperative recurrence‐free survival (RFS) and overall survival (OS) on the basis of the tumor stage and extent of nodal metastases among patients with T2 disease.

### 2.1 Statistics

Statistical analyses were performed using IBM SPSS Statistics version 23 (IBM Corp., Armonk, NY, USA). Continuous data are expressed as median and range and were compared using the Mann–Whitney *U*‐test. Categorical data were compared by Pearson's chi‐squared test. RFS was calculated from the date of surgical resection to the date of first recurrence, and only patients alive without tumor recurrence at the last follow‐up date were censored. OS was calculated from the date of surgery to death from any cause and censored on the date of the last follow‐up. Survival curves were constructed by the Kaplan–Meier method and compared with the results of the log‐rank test. Hazard ratios were calculated using a Cox proportional hazard model. *p* < .05 was considered statistically significant for all analyses.

## 3 RESULTS

### 3.1 Patient selection

The database included 596 patients with pathologically confirmed GBCA. The number of patients in each of the six institutions (named as #1 to #6) was #1, 178; #2, 94; #3, 128; #4, 80; #5, 63; and #6, 53. This included 64 patients with cystic duct carcinoma in which the main tumor site was in the cystic duct. Among the 596 patients, 46 patients who underwent only exploratory laparotomy with advanced or metastatic disease and 13 patients with insufficient data to determine the TNM stage were excluded. Among the remaining 537 patients, the median TLNC was 10. TLNC was <6 or not recorded in 158 patients. The surgical procedure for these 158 patients was mainly cholecystectomy with gallbladder bed resection (*n* = 71) followed by cholecystectomy (*n* = 61). After excluding these patients, the remaining 379 patients (institution #1, 128; #2, 72; #3, 71; #4, 41; #5, 42; and #6, 25) were selected for the analyses in this study.

Table 1 shows the comparison of perioperative profiles and distributions of pTNM stagings between the early (2002–2012) and late (2013–2022) period. Most factors were comparable except the difference in types of surgical resection with decrease in the proportion of pancreaticoduodenectomy and length of postoperative hospital stay. The most common procedure in patients with pT2 GBCA was cholecystectomy with GB bed resection, with preservation of the extrahepatic bile duct (*n* = 93, 57.1%) or with resection of the extrahepatic bile duct (*n* = 21, 12.9%) followed by pancreaticoduodenectomy (*n* = 20, 12.2%). In comparison, in patients with pT3/4 disease, major hepatectomy (≥3 Couinaud segments) was the most common procedure (*n* = 116, 60.7%), and in 30 patients, major hepatectomy with pancreaticoduodenectomy was performed. The numbers of laparoscopic surgery were eight for cholecystectomy with GB bed resection, with preservation of the extrahepatic bile duct, one for cholecystectomy, and one for cholecystectomy with GB bed and the extrahepatic bile duct resection.

**TABLE 1:** Comparison of perioperative characteristics between the early (2002–2012) and late (2013–2022) period.

Variable	Total	2002–2012	2013–2022	*p* (between the two periods)
	*n* = 379	*n* = 140	*n* = 239	
Age [years, median (range)]	71 (40–89)	71 (40–87)	71 (44–89)	.362
Sex, male (%)	208 (54.9%)	76 (54.3%)	132 (55.2%)	.859
Preoperative chemotherapy performed	18 (4.7%)	3 (2.1%)	15 (6.3%)	.068
CEA (ng/mL)	2.6 (0.3–333.8)	2.6 (0.5–333.8)	2.5 (0.3–301)	.090
CA19‐9 (U/mL)	23.6 (0.4–162 017)	31 (1–46 660)	23 (0.4–162 017)	.320
Surgical procedure				
Type of resection				
GB	13 (3.4%)	4 (2.9%)	9 (3.8%)	.011
eGB	149 (39.3%)	57 (40.7%)	92 (38.5%)	
eGB + BD	37 (9.8%)	6 (4.3%)	31 (13.0%)	
Major Hx	102 (26.9%)	33 (23.6%)	69 (28.9%)	
PD ± eGB	41 (10.8%)	21 (15.0%)	20 (8.4%)	
PD + major Hx	37 (9.8%)	19 (13.6%)	18 (7.5%)	
Vascular resection	41 (10.8%)	18 (12.9%)	23 (9.6%)	.328
Other organ resection	14 (3.7%)	7 (5.0%)	7 (2.9%)	.302
R0 resection	313 (82.6%)	118 (84.3%)	195 (81.6%)	.504
Postoperative LOS (days)	21 (3–153)	23 (3–147)	18.5 (5–153)	.027
90‐day mortality	9 (2.4%)	2 (1.4%)	7 (2.9%)	.355
Postoperative adjuvant chemotherapy performed	26 (6.9%)	8 (5.7%)	18 (7.5%)	.499
T				
T1	29 (7.7%)	8 (5.7%)	21 (8.8%)	.194
T2	162 (42.7%)	63 (45.0%)	99 (41.4%)	
T3	141 (37.2%)	57 (40.7%)	84 (35.2%)	
T4	47 (12.4%)	12 (8.6%)	35 (14.6%)	
N				
N0	190 (50.1%)	72 (51.4%)	118 (49.4%)	.926
N1	145 (38.3%)	52 (37.1%)	93 (38.9%)	
N2	44 (11.6%)	16 (11.4%)	28 (11.7%)	
M				
M0	334 (89.5%)	128 (92.8%)	206 (87.7%)	.121
M1	39 (10.5%)	10 (7.3%)	29 (12.3%)	

Abbreviations: CEA, carcinoembryonic antigen; CA19‐9, carbohydrate antigen 19‐9; GB, cholecystectomy; eGB, extended cholecystectomy with gallbladder bed resection up to segment 4B and 5; BD, extrehepatic bile duce resection; PD, pancreaticoduodenectomy; major Hx, major hepatectomy of ≥3 Couinaud segments; LOS, length of hospital stay; T, tumor stage; N, lymph node stage; M, metastasis.

### 3.2 Number and extent of metastatic nodes by the pT stage

**FIGURE 2:**
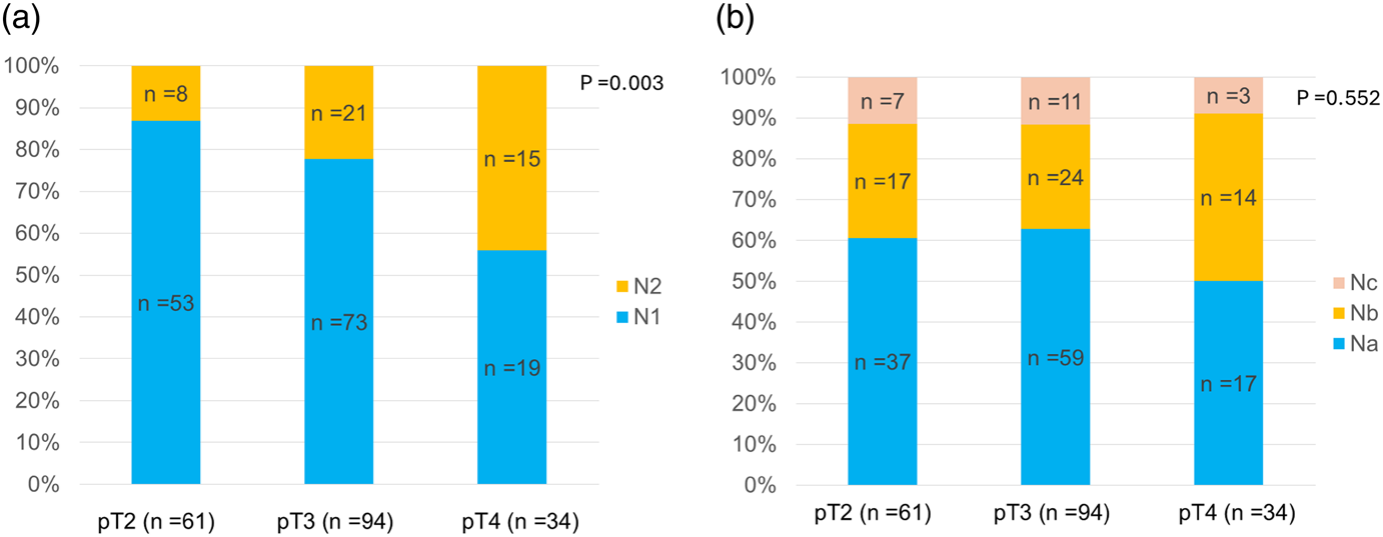
Proportions of patients with N1 and N2 (a) or Na, Nb, and Nc (b) disease in each pT category among patients with node‐positive disease. N, lymph node; pT, pathological T stage.

There were no patients with pT1 node‐positive disease. The proportion of patients with node‐positive disease increased in accordance with the pT category (pT1, 0/29 [0%]; pT2, 62/163 [38.0%]; pT3, 96/143 [67.1%]; pT4, 35/48 [72.9%]; *p* < .001). The presence of node metastases was associated with the histological tumor type (papillary or intracholecystic neoplasm, 11/38 [29%]; well differentiated adenocarcinoma, 41/101 [41%]; others, 136/237 [57%]; *p* < .001). Among 231 patients with the records on tumor sites, the node metastases were more frequent in the patients with tumors involving the gallbladder neck or cystic duct (81/141, 57%) than those with tumors limited to the gallbladder body or fundus (33/90, 37%) (*p* = .002). Figure 2 shows the proportions of patients with N1 and N2 (Figure 2a) disease and the proportions for Na, Nb, and Nc disease (Figure 2b) in each pT category among the patients with node‐positive disease. The metastatic nodes in the patients with Nc were para‐aortic nodes in 10, nodes along the celiac axis in 5, nodes along the celiac axis and para‐aortic node in 2 patients, and nodes along the right gastric artery, along the right gastroepiploic artery, along the splenic artery, and along the right gastroepiploic artery and middle colic artery in one patient, respectively. The proportion of patients with N2 disease increased with increasing T grade, while the proportions of patients with Na, Nb, and Nc disease were comparable between the three pT categories. The proportion of patients with Nb/c disease among those with node‐positive disease increased as the number of metastatic nodes increased in both pT2 disease (solitary node, 3/26 [11.5%]; 2 nodes, 8/17 [47.0%]; 3 nodes, 6/10 [60.0%]; ≥4 nodes, 7/8 [87.5%]; *p* < .001) and pT3/4 disease (solitary node, 7/48 [14.6%]; 2 nodes, 9/26 [34.6%]; 3 nodes, 9/18 [50.0%]; ≥4 nodes, 27/36 [75.0%]; *p* < .001). Among the patients with pT2 GBCA, the proportion of patients with Nb and Nc increased according to the number of positive nodes except for the patients with three lymph node metastases, and 50% (4/8) patients with ≥4 positive nodes were categorized as Nc (Table S1).

### 3.3 Incidence of metastases skipping lymph nodes along the cystic duct (#12c) or region A lymph nodes

**FIGURE 3:**
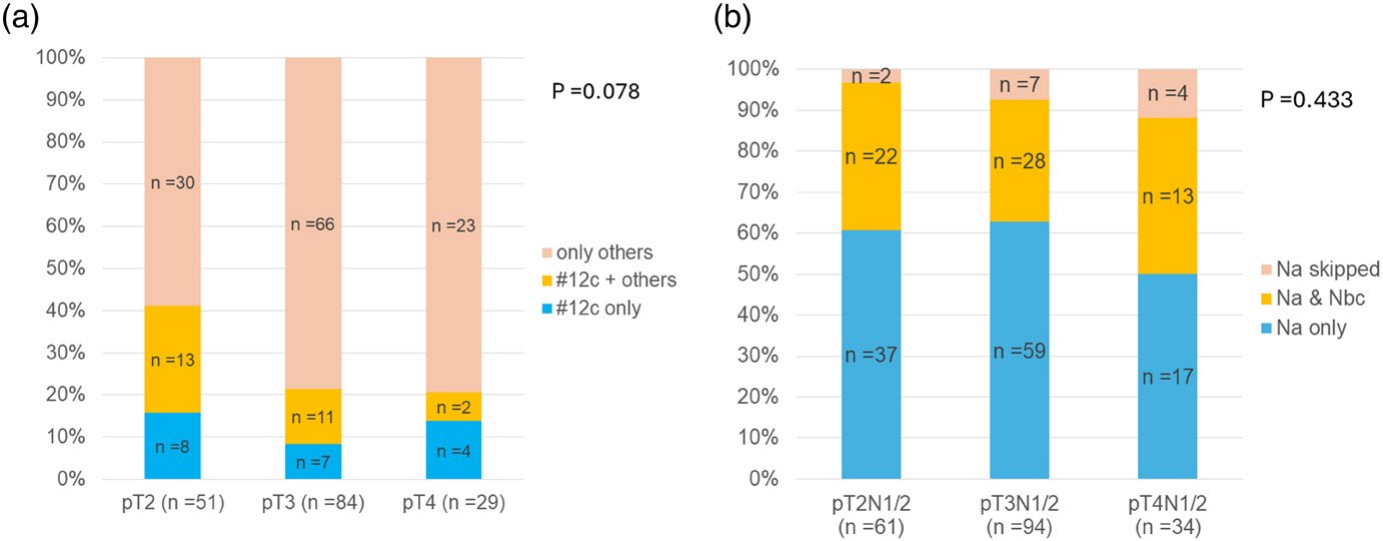
(a) Incidence of node metastases limited to #12c (cystic nodes), #12c with others, and only others skipping #12c. (b) Incidence of node metastases limited to region A nodes, region A and others in region B or C, and only region B or C nodes skipping region A.

Among the patents with node‐positive disease, the incidence of metastases limited to #12c, metastases to #12c and others, and only nodes other than #12c was 8 patients (15.6%), 13 patients (25.4%), and 30 patients (58.8%) for pT2 disease; 7 patients (8.3%), 11 patients (13.1%), and 66 patients (78.5%) for pT3 disease; and 4 patients (13.8%), 2 patients (6.9%), and 23 patients (79.3%) for pT4 disease, respectively (*p* = .078) (Figure 3a). The incidence of metastases skipping #12c was significantly higher in patients with pT3/4 disease compared with that in patients with pT2 disease (89/113 [78.8%] vs. 30/51 [58.8%], *p* = .008). In comparison, the incidence of metastases limited to region A nodes, metastases to region A and region B/C nodes, and only region B/C nodes was 37 patients (60.7%), 22 patients (36.1%), and 2 patients (3.3%) for pT2 disease; 59 patients (62.8%), 28 patients (29.8%), and 7 patients (7.4%) for pT3 disease; and 17 patients (50.0%), 13 patients (38.2%), and 4 patients (11.8%) for pT4 disease, respectively (*p* = .433) (Figure 3b). The incidence of metastases that skipped region A nodes was comparable between pT2 (2/61 [3.3%]) and pT3/4 disease (11/128 [8.6%]) (*p* = .177).

### 3.4 Survival outcomes

**FIGURE 4:**
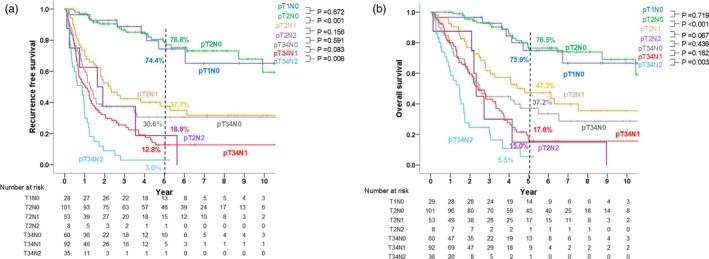
Recurrence‐free (a) and overall survival curves (b) for patients with pT1N0, pT2N0, pT2N1, pT2N2, pT3/4N0, pT3/4N1, and pT3/4N2 gallbladder cancer.

Figure 4 shows the RFS and OS for all patients. Both RFS and OS for patients with pT2N1 disease (5‐year RFS, 37.7%; 5‐year OS, 47.3%) and those with pT2N2 (5‐year RFS, 18.8%; 5‐year OS, 15.0%) were significantly worse compared with those for patients with pT2N0 disease (5‐year RFS, 78.8%, 5‐year OS, 76.5%,) and comparable to those with pT3/4N0 disease (5‐year RFS, 30.6%, 5‐year OS 37.2%). The hazard ratio for OS for pT2N1 or pT2N2 to pT2N0 disease was 3.104 (95% confidence interval [CI], 1.760–5.475) or 8.233 (95% CI, 3.387–20.015), respectively, and that of pT34N1 or pT34/N2 to pT34/N0 disease was 1.337 (95% CI, 0.889–2.011) and 2.348 (95% CI, 1.422–3.879), respectively.

R0 resection rates in the patients with pT1, pT2, pT3, and pT4 disease were 96.6% (28/29), 87.7% (142/162), 80.1% (113/141), and 63.8% (30/47), respectively. The survival curves among these 313 patients with R0 resection were presented in Figure S1. Similarly to the results with the whole patient population, the RFS and OS of the patients with pT2N1 or pT2N2 disease were comparable to those of the patients with pT34N0 disease.

**FIGURE 5:**
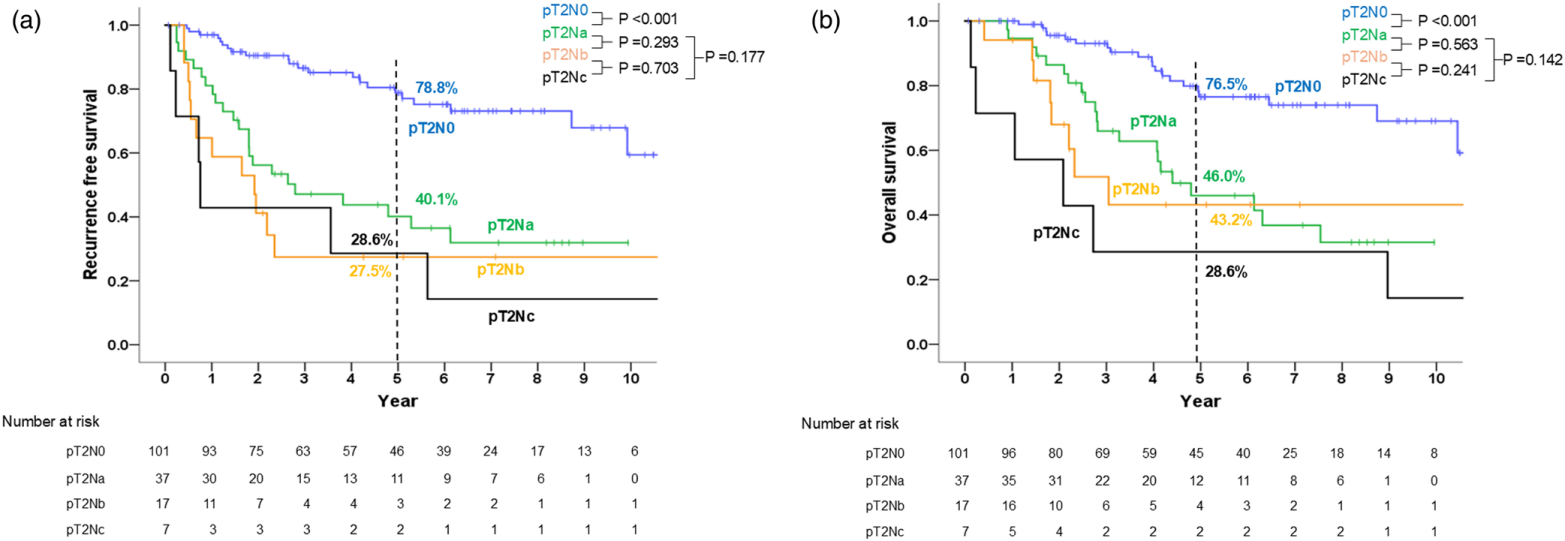
Comparison of recurrence‐free‐ (a) and overall survival (b) between patients with pT2Na, pT2Nb, and pT2Nc gallbladder cancer.

Figure 5 shows the comparison of the survival curves between Na, Nb, and Nc status among the patients with pT2 disease. Their perioperative profiles were summarized in Table S2. In these patients, RFS (*p* = .320) and OS (*p* = .293) were comparable between the three groups. Furthermore, patients with node metastases limited to #12c, outside of #12c but limited to region A, and outside of region A had comparable RFS and OS (5‐year RFS, 37.5% vs. 41.1% vs. 27.8%, respectively [*p* = .258]; 5‐year OS, 62.5% vs. 41.5% vs. 38.8%, respectively [*p* = .536]). The comparison of survival curves between Na, Nb, and Nc status among the patients with pT3/4 disease were shown in Figure S2, showing significant difference only between those with Na and Nb disease.

## 4 DISCUSSION

The present study evaluated the N status for both the number and location of metastatic nodes. Generally, the former is simpler, and several studies have shown the utility of the number or proportion of metastatic nodes as a prognostic predictor.^16^, ^17^, ^18^ The incidence and number of lymph node metastases increased with increasing pT stage in this study. In patients with pT2 disease, the proportion with node metastases was 38%, and the metastasis was solitary in nearly half of these patients. In comparison, the extent of metastases on the basis of the distributions of the three region groups (A, B, and C) was comparable between pT2, pT3, and pT4 among patients with node‐positive disease. Our results showing region B/C node metastases even in patients with N1 disease suggest that a small number of node metastases do not necessarily mean localized metastases for both pT2 and pT3/4 GBCA. In pT2 disease, the proportion of patients with extensive node metastases increased with the number of metastatic nodes. Notably, the incidence of region B or C metastases was approximately 50% even in patients with metastases to two or three nodes.

The present study also showed that node metastases skipping the cystic node (#12c) were frequent irrespective of pT status. Because 25 patients (pT2, 10; pT3, 10; and pT4, 5) were excluded from the analysis presented in Figure 3a because they lacked accurate data to specify if the positive nodes were #12c or other nodes along the hepatoduodenal ligament, the estimation might not be completely precise. However, our results suggested that the commonly used term, “sentinel node” for #12c is misleading. Several studies to date have similarly shown that metastases skipping the cystic node were common. Kokudo et al. showed that among 37 patients with metastases to group 2 nodes (metastases to the pericholedochal nodes, and nodes along the proper hepatic artery and portal vein), #12c nodes were free from cancer in 29% of the patients.^19^ In Yasukawa et al.'s study of 80 patients with T2 and T3 GBCA, node metastases skipping cystic duct nodes did not occur in T2a disease (T2 tumor on the peritoneal side, 0/18 [0%]) and occurred only in T2b disease (tumor on the hepatic side, 3/20 [15%]) and T3 disease (2/22 [9%] in patients with a T3 tumor on the peritoneal side, and 11/20 [55%] in those with a tumor on the hepatic side).^13^ In comparison, the incidence of metastases skipping region A nodes was low. In a previous study by Birnbaum et al. of 112 patients with GBCA, the incidence of metastases skipping the nodes in the hepatic pedicle was 23% (5/22), although the incidence in those specifically with T2 disease was not presented.^20^

We would like to emphasize the importance of our study in selecting patients on the basis of the TLNC with pathological evaluation. To the best of our knowledge, our study evaluated the largest number of patients with TLNC ≥6. However, whether the cut‐off of 6 nodes is appropriate is controversial. Kim et al. insisted that TLNC ≥8 is expected to ensure accurate staging of T3 GBCA.^21^ At least, selecting patients on the basis of TLNC enhanced the quality of our study and enabled accurate evaluation of the prognosis of “true” N0 patients by preventing the underestimation of node metastases.

Generally, a GBCA tumor extending out of the gallbladder serosa is classified as T3 or T4 disease. The postoperative prognosis of T3N0 disease is reportedly worse than that of T2N1/2 disease.^22^, ^23^ Therefore, the impact of these local extensions on prognosis would be stronger than that of lymph node metastases. However, the results of the present study showed that the prognosis of patients with pT2N1/2 and pT3/4N0 disease was comparable. This suggests that the impact of node metastases in pT2 GBCA is equivalent to that of extraserosal tumor extension. Additionally, the hazard ratio of postoperative survival for nodal metastases was higher in pT2 GBCA compared with that in pT3/4 GBCA. Notably, our results revealed that patients with pT2 GBCA with metastases “limited to cystic nodes” had a comparable prognosis to that of patients with more extensive node metastases. Among the various types of tumor extension of GBCA that are mainly classified by the direction of tumor invasion, such as gallbladder bed and/or hepatic hilum,^24^, ^25^ Kondo et al. named one category as “lymph node type.” The authors explained this type as follows: “although the primary tumor is seldom large, enlarged metastatic lymph nodes in the hepatoduodenal ligament around the head of the pancreas and frequently the para‐aortic area are the most prominent feature.” The authors classified 15 of 112 patients as having this type and reported a 5‐year postoperative survival rate of 7%.^24^ To date, no follow‐up studies have been performed to evaluate the incidence or prognosis of this type of disease. In our study, T2 Nb/c disease, which accounted for 15% and 39% of the patients with pT2 and pT2N1/2 disease, respectively, could possibly constitute the lymph node type described by Kondo et al., suggesting this type was not at all uncommon.

Although our results showed a significantly worse prognosis for patients with pT2N1/2 disease compared with that for patients with T2N0 disease, this does not deny the surgical indication for this group of patients. In Higuchi et al.'s series, 5‐year disease‐specific survival of patients with T2 GBCA and metastases to posterosuperior pancreatic head nodes (13 patients) or the nodes along the superior mesenteric artery (2 patients), corresponding to our Nb or Nc stages, respectively, was 91.7% and 50%, respectively.^9^ Recently, the same group showed that the prognosis of patients with positive posterosuperior pancreatic head nodes with 1–3 node metastases was comparable to that of patients with N1 disease and better than that of patients with more distant node metastases.^26^ Additionally, a study by Aggarwal et al. showed that selected patients with para‐aortic lymph node metastases achieved a meaningful prognosis, with a median postoperative survival of 20 months.^27^ In contrast to the previous studies showing better survival in the patients with posterosuperior pancreatic head nodes than those with metastases to more distant nodes,^7^, ^8^ the prognosis of patients with Nb disease was comparable to those with Na or Nc disease in our series. The small number of patients in each group and the bias that only the patients expected to show long survival irrespective of Nb or Nc disease underwent surgical resection could have affected the results. In fact, 5 of 24 patients (21%) with pT2Nb/c disease, including two with para‐aortic lymph node metastases, survived more than 5 years in our series. The present study is notable, however, to show that in pT2 GBCA, any lymph node metastases irrespective of the sites were associated with worse prognosis compared with those without node metastases and should be considered a prognostic factor. In the present series, postoperative adjuvant chemotherapy was performed only rarely. Only seven patients (11%) with pT2N1/2 disease received postoperative adjuvant chemotherapy. Therefore, we hypothesize that it would be feasible to consider adjuvant therapy for all patients with node‐positive disease. On the contrary, it is difficult to propose the appropriate extent of lymph node dissection based on the results of this study.

The limitations of this study include selection bias owing to the retrospective study design. The extent of lymph node dissection was basically regions A and B, as described in the Methods section, but the actual extent of node dissection depended on the strategy of each institution and might not be consistent. Instead, the exclusion of patients with TLNC <6 would have compensated for the selection bias or underestimation of node metastases due to the ununited nodal dissection. A preliminary survey with the participating six institutions showed that, in this era that multidisciplinary treatment is considered beneficial; chemotherapy was generally preferred as the first‐choice treatment for localized GBCA with suspicious metastases to the posterior superior pancreatic head lymph nodes. However, a comparison of outcomes in patients with pT2N1/2 disease who received chemotherapy was not performed. Additionally, evaluation of the role of preoperative chemotherapy, which was rarely performed in the present series, is needed. Several randomized controlled trials suggested the survival benefit of postoperative adjuvant chemotherapy in patients with biliary tract cancer.^28^, ^29^ However, the proportion of patients with GBCA enrolled in these studies was small, and another randomized controlled trial comparing adjuvant six cycles of gemcitabine and cisplatin therapy with observation for patients with GBCA, with 50 patients in each arm, failed to show improved disease‐free or overall survival.^30^ Larger studies to evaluate the benefit of perioperative chemotherapy, including specifically the patients with GBCA, are expected. In addition, we did not differentiate between T2a and T2b disease, which was first proposed in the eighth edition of the UICC guidelines^2^ based on a study showing worse survival for T2 GBCA located on the hepatic side.^31^ Toge et al. compared T2a and T2b GBCA and showed a significantly higher incidence of regional lymph node metastases and worse prognosis in patients with T2b versus T2a disease. However, among patients with node metastases, the number and extent of metastatic nodes were comparable. The authors proposed that the extent of node dissection should not be changed on the basis of tumor location.^10^

To summarize, the present study showed that lymph node metastases were recognized in 38% of the patients with pT2 GBCA. In most of these patients, the number of metastases was less than four; however, metastases skipping the #12c nodes to beyond the hepatoduodenal ligament were common. pT2N1/2 GBCA showed a comparable prognosis to that of T3/4N0 GBCA. In conclusion, pT2N12 GBCA should be considered a type of advanced disease with a comparable prognosis to that of locally advanced disease irrespective of the number or location of metastatic nodes.

## FUNDING INFORMATION

This work was supported by the Foundation for the Promotion of Defense Medicine.

## CONFLICT OF INTEREST STATEMENT

The authors declare no conflict of interest for this article.

## Supporting information


Figure S1.
Figure S2.

Table S1.
Table S2.
