# Supplementary figures and images for: Comparable impact of lymph node metastases in T2 gallbladder cancer on postoperative prognosis irrespective of the extent of the metastases: A retrospective analysis
Source: J Hepatobiliary Pancreat Sci. 2025 Mar 24;32(6):443–51. doi: 10.1002/jhbp.12140 (PMC12188152; doi:10.1002/jhbp.12140)

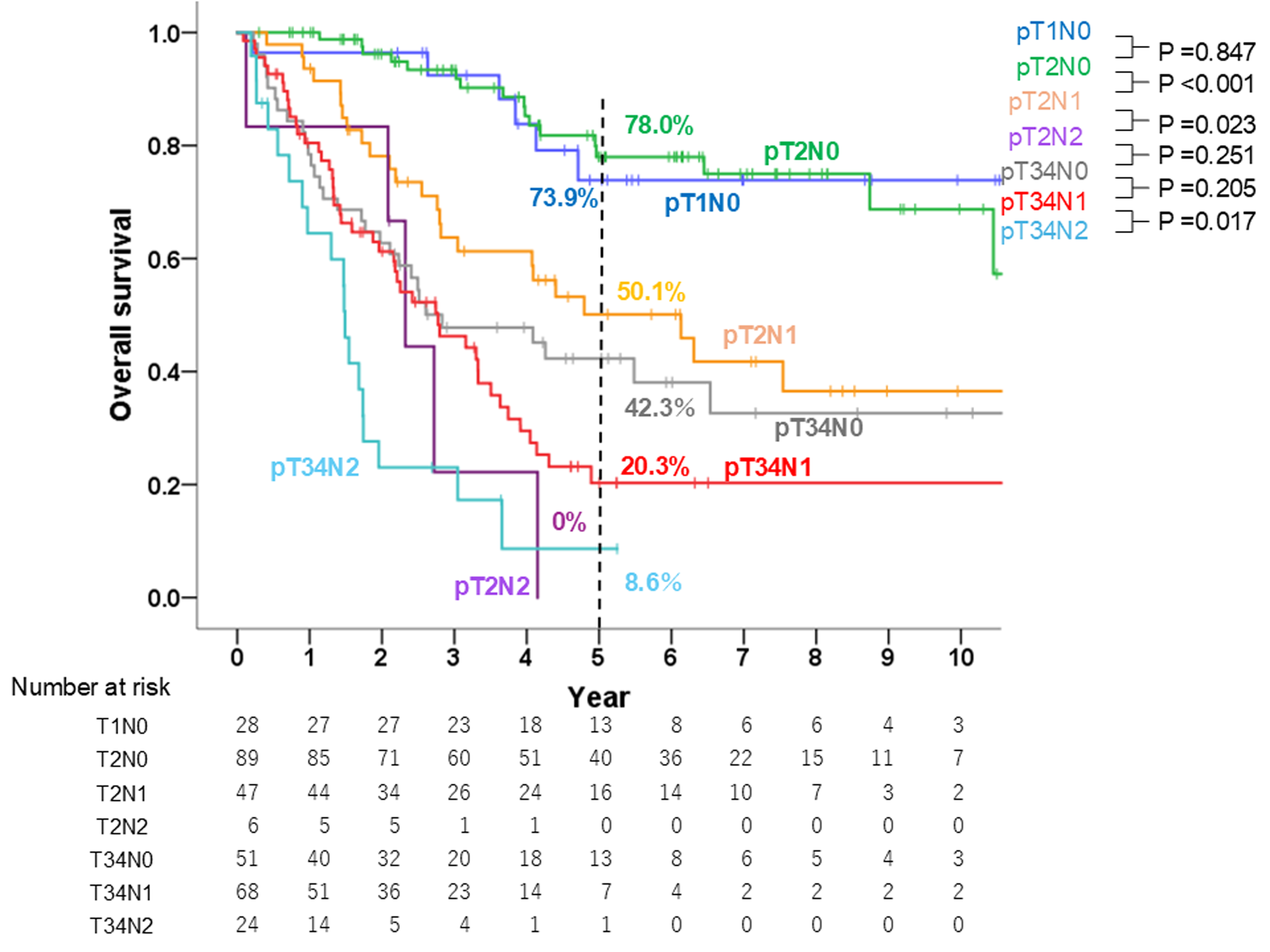

Supplement: Supplementary file 1 — Figure S1. Figure S2. [file JHBP-32-443-s002.zip › jhbp12140-sup-0003-supinfo_Supple Fig 1b.tif]

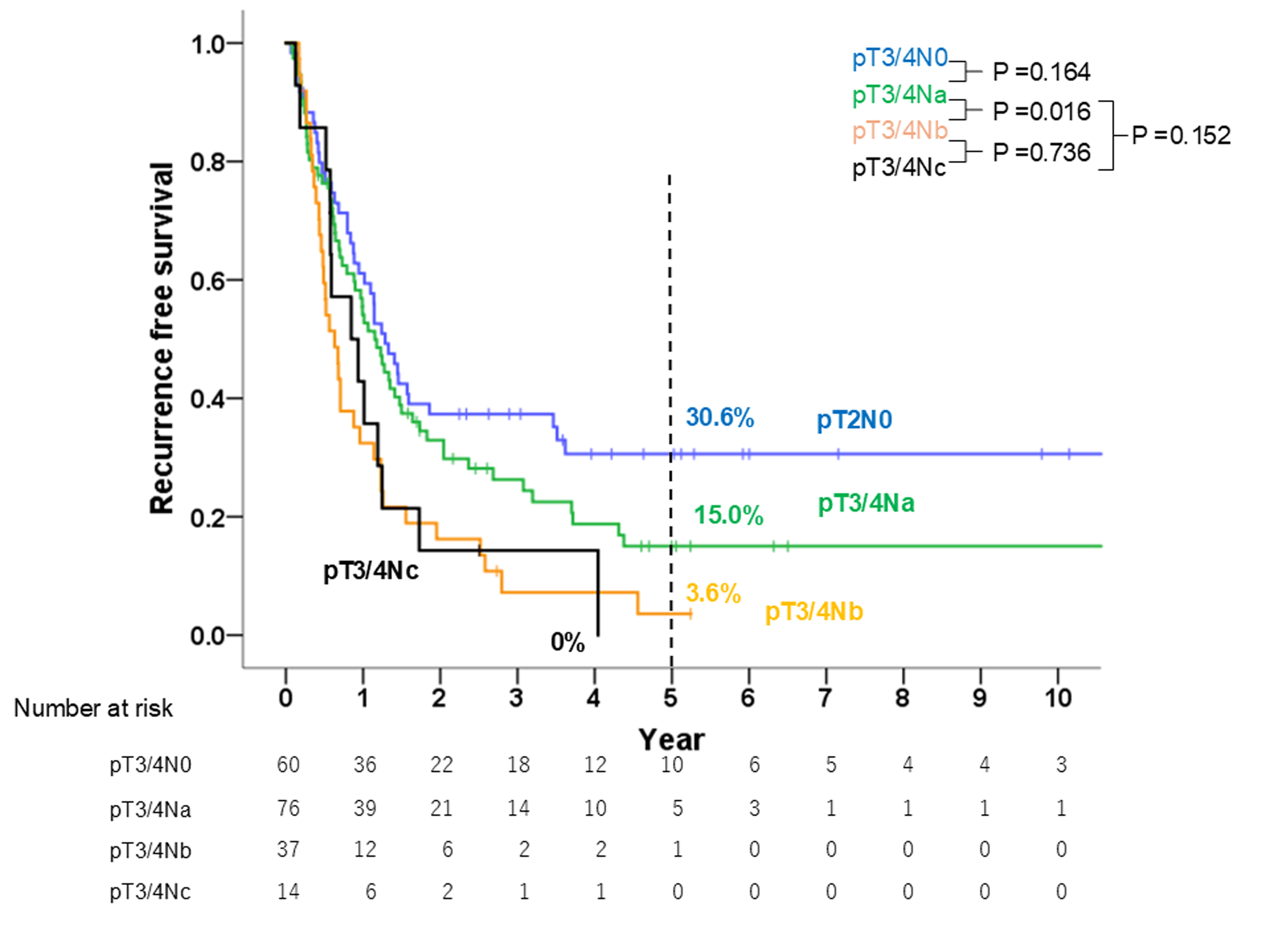

Supplement: Supplementary file 1 — Figure S1. Figure S2. [file JHBP-32-443-s002.zip › jhbp12140-sup-0004-supinfo_Supple Fig 2a.tif]

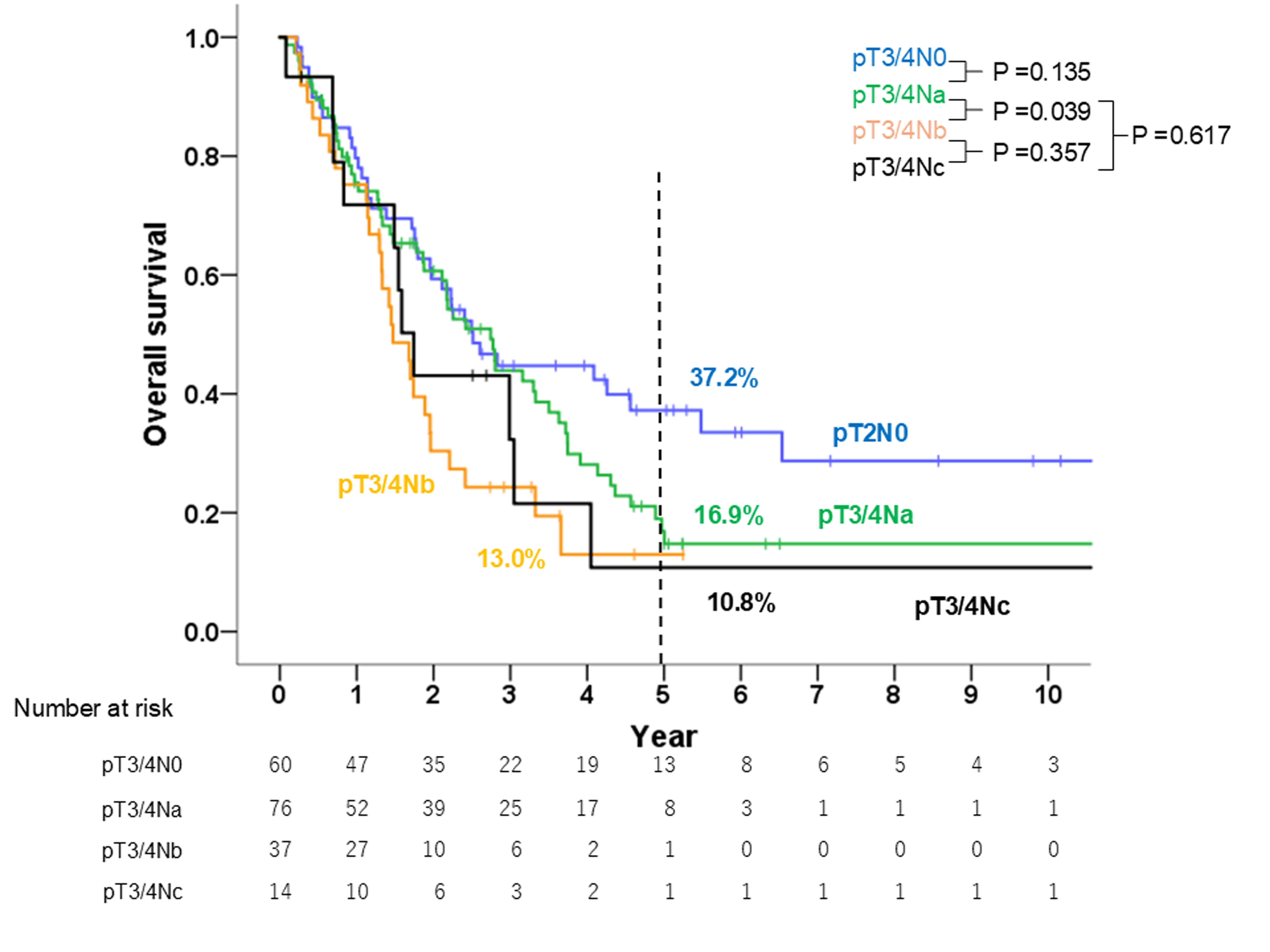

Supplement: Supplementary file 1 — Figure S1. Figure S2. [file JHBP-32-443-s002.zip › jhbp12140-sup-0005-supinfo_Supple Fig 2b.tif]

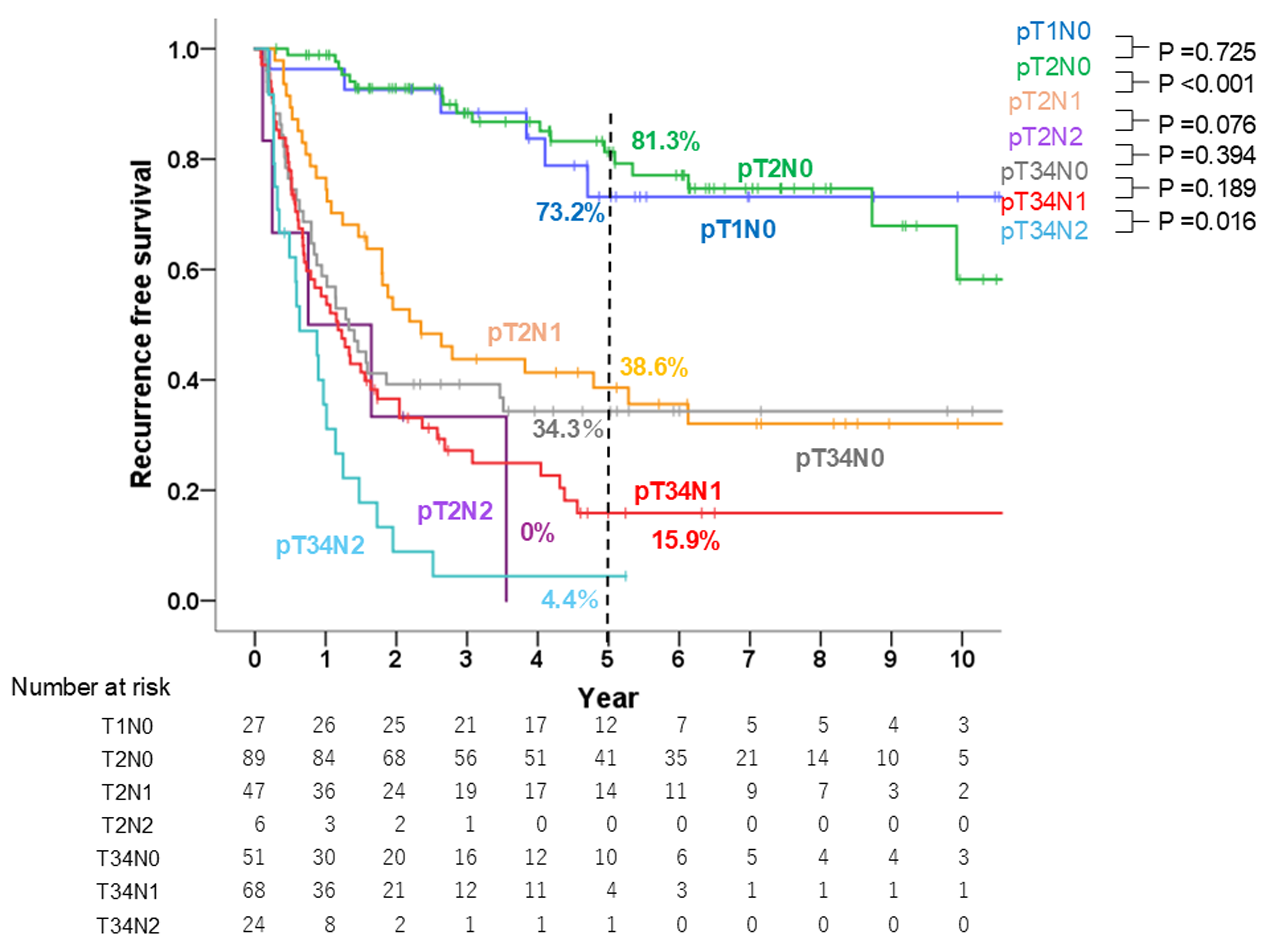

Supplement: Supplementary file 1 — Figure S1. Figure S2. [file JHBP-32-443-s002.zip › jhbp12140-sup-0002-supinfo_Supple Fig 1a.tif]
