# Supplementary material for: Comparable impact of lymph node metastases in T2 gallbladder cancer on postoperative prognosis irrespective of the extent of the metastases: A retrospective analysis
Source: J Hepatobiliary Pancreat Sci. 2025 Mar 24;32(6):443–51. doi: 10.1002/jhbp.12140 (PMC12188152; doi:10.1002/jhbp.12140)
Supplement: Supplementary file 2 — Table S1. Table S2. [file JHBP-32-443-s001.docx]

**Supplementary Table 1.** Extent of node metastases according to the number of positive nodes in pT2 gallbladder cancer

| Number of positive nodes | Extent of metastatic nodes | | | Total |
| --- | --- | --- | --- | --- |
|  | Na | Nb | Nc |  |
| 1 | 23 (88%) | 2 (8%) | 1 (4%) | 26 |
| 2 | 9 (53%) | 5 (29%) | 3 (18%) | 17 |
| 3 | 4 (40%) | 6 (60%) | 0 | 10 |
| ≥4 | 1 (13%) | 3 (38%) | 4 (50%) | 8 |

**Supplementary Table 2.** Comparison of perioperative profiles between the patients with pT2Na, pT2Nb, and pT2Nc

| **Variable** | | | **pT2Na**  **(N =37)** | **pT2Nb**  **(N =17)** | **pT2N2c**  **(N =7)** | **P** |
| --- | --- | --- | --- | --- | --- | --- |
| Age (years, median (range)) | | | 73 (50-83) | 77 (40-84) | 73 (46-89) | 0.4311 |
| Sex, Male (%) | | | 17 (46%) | 8 (47%) | 5 (71%) | 0.4557 |
| Preoperative chemotherapy performed | | | 0 | 1 (6%) | 0 | 0.2683 |
| CEA (ng/ml) | | | 3.2 (0.6-22.1) | 2.8 (1.4-225) | 5 (1.5-5.9) | 0.8053 |
| CA19-9 (U/ml) | | | 17 (1-1280) | 20 (1-1577) | 153 (1.7-1710) | 0.2131 |
| Surgical procedure | Type of resection | GB | 2 (5%) | 1 (6%) | 1 (14%) | 0.2949 |
|  |  | eGB | 19 (51%) | 7 (41%) | 2 (29%) |  |
|  |  | eGB + BD | 1 (3%) | 4 (24%) | 0 |  |
|  |  | Major Hx | 4 (11%) | 1 (6%) | 1 (14%) |  |
|  |  | PD ± eGB | 8 (22%) | 3 (18%) | 1 (14%) |  |
|  |  | PD + Major Hx | 3 (8%) | 1 (6%) | 2 (29%) |  |
|  | Vascular resection | | 1 (3%) | 1 (6%) | 1 (14%) | 0.4198 |
|  | Other organ resection | | 0 | 0 | 0 | N.A. |
| R0 resection | | | 33 (89%) | 15 (88%) | 5 (71%) | 0.4345 |
| Postoperative LOS (days) | | | 22 (8-80) | 15 (8-68) | 24 (8-60) | 0.5718 |
| 90-day mortality | | | 0 | 0 | 2 (29%) | 0.0003 |
| Postoperative adjuvant chemotherapy performed | | | 2 (5%) | 2 (12%) | 2 (29%) | 0.1603 |
| Histology | Pap/ICPN | | 5 (14%) | 3 (18%) | 0 | 0.8034 |
|  | Well | | 16 (43%) | 6 (35%) | 3 (43%) |  |
|  | Other | | 16 (43%) | 8 (47%) | 4 (57%) |  |
| pN | N1 | | 36 (97%) | 14 (82%) | 3 (43%) | 0.0004 |
|  | N2 | | 1 (3%) | 3 (18%) | 4 (57%) |  |
| pM | M0 | | 36 (97%) | 13 (76%) | 3 (43%) | 0.0005 |
|  | M1 | | 1 (3%) | 4 (24%) | 4 (57%) |  |

Footnotes: Abbreviations: GB, cholecystectomy; eGB, extended cholecystectomy with gallbladder bed resection up to segment 4B and 5; BD, extrahepatic bile duct resection; PD, pancreaticoduodenectomy; Major Hx, major hepatectomy of ≥ 3 Couinaud segments; CEA, carcinoembryonic antigen; CA19-9, carbohydrate antigen 19-9; LOS, length of hospital stay;Pap, papillary adenocarcinoma; ICPN, intracholecystic papillary neoplasm; Well, well differentiated adenocarcinoma; N, lymph node stage; M: metastasis.
